# Editorial: MOGAD, current knowledge and future trends

**DOI:** 10.3389/fneur.2023.1283217

**Published:** 2023-09-28

**Authors:** Sasitorn Siritho, Lekha Pandit, Marcelo Matiello

**Affiliations:** ^1^Bumrungrad International Hospital, Bangkok, Thailand; ^2^Faculty of Medicine, Siriraj Hospital, Mahidol University, Bangkok, Thailand; ^3^K. S. Hegde Medical Academy, Mangaluru, Karnataka, India; ^4^NITTE University Center for Science Education and Research (NUCSER), Mangaluru, Karnataka, India; ^5^Massachusetts General Hospital, Harvard Medical School, Boston, MA, United States

**Keywords:** editorial, MOGAD, current, knowledge, trends

In the ever-evolving landscape of neuroimmunology, both basic and clinical research, one condition has captured the attention of researchers, clinicians, and patients alike: Myelin Oligodendrocyte Antibody Disease (MOGAD). This enigmatic disorder, characterized by its distinctive serum biomarker and severe central nervous system (CNS) inflammatory attacks, warrants an in-depth exploration of its current understanding and the potential trends shaping its future trajectory.

Similar to Neuromyelitis Optica Spectrum Disorder (NMOSD), MOGAD resides at the intersection of autoimmune disorders and neurology, challenging conventional notions within the realm of demyelinating diseases. Recent advancements in diagnostic tools, particularly the development of sensitive and specific tests for detecting anti-MOG antibodies, have revolutionized our ability to identify and differentiate MOGAD from other similar conditions, such as multiple sclerosis (1, 2). The presence of high titers of this serum antibody in patients with neurological disorders is now a diagnostic cornerstone (3). This heightened accuracy has expanded the spectrum of known clinical manifestations, transcending the common occurrences of optic neuritis, transverse myelitis, and ADEM-like presentations (4). Consequently, this diagnostic precision has led to the formulation of recent diagnostic criteria and, most importantly, earlier diagnoses and more targeted treatment approaches, ultimately enhancing patient outcomes.

In this Research Topic of *Frontiers in Neurology*, we called for original work on the theme of “*MOGAD, Current Knowledge and Future Trends*” In addition to original research on MOGAD. This Research Topic also called for review to collect novel approaches that may help clarify this unique condition.

## Refining diagnosis, epidemiology and expanding the clinical spectrum

In this Frontiers in Neurology Research Topic, Lang et al. compared optical coherence tomography (OCT)/OCT angiography (OCTA) measures in patients with NMOSD and MOGAD, revealing that MOGAD patients exhibit reduced superficial vascular plexus density. This suggests distinct pathological processes and underlying macular structural and microvascular impairments. Li et al. described the clinical profiles and treatment responses of a cohort of 93 Chinese patients with pediatric MOGAD. Smith et al. reported excellent agreement between fixed and live CBA for MOG antibody testing in a real-world clinical cohort of 322 serum samples. Xu et al. documented two cases of cortical encephalitis with high titers of MOG antibodies. Perez-Giraldo et al. reviewed typical and atypical features of transverse myelitis in MOGAD patients. Hor and Fujihara conducted a global review of MOGAD epidemiology, consolidating current knowledge. Vlad et al. analyzed cerebrospinal fluid parameters in 30 adult MOGAD patients and 189 adult patients with Relapsing-Remitting Multiple Sclerosis (RRMS), revealing higher mean QAlb values and a lower presence of OCBs in the MOGAD cohort.

## Unraveling the underlying mechanisms

Ongoing research focuses on unraveling the intricate mechanisms driving MOGAD. Scientists are keen to dissect the immune responses involved and decipher the factors triggering antibody attacks on myelin. By doing so, they aim to uncover novel therapeutic targets and interventions. Corbali and Chitnis extensively reviewed the immunological aspects of MOGAD pathophysiology, emphasizing the activation of T cells in the periphery followed by reactivation in the subarachnoid/perivascular spaces by MOG-laden antigen-presenting cells. They also highlighted that abnormal levels of neuroinflammatory biomarkers in MOGAD suggest that most axonal damage occurs during the initial attack. Takai et al. provided a detailed analysis of lesion pathology in MOGAD, noting that perivenous complement deposition is less common than in NMOSD but is observed in myelinated fibers and on myelin degradation products within macrophages. Malli et al. reported a lower frequency of H. Pylori infection in MOGAD and MS patients compared to NMOSD patients, hypothesizing that this infection might serve as a marker of gut hygiene and the onset of autoimmunity. Trentinaglia et al. studied a cohort of 150 MOGAD patients, revealing only two cases with concomitant cancer. They also reviewed an additional 15 case reports in the literature, concluding that MOGAD is unusually associated with a paraneoplastic syndrome. Oertel et al. reviewed appropriate animal models for translational MOGAD research and the current state and prospects of translational research imaging in this disease.

While the progress in MOGAD research is promising, significant challenges remain. Variability in clinical presentations, the complex interplay of immune responses, and the necessity for long-term clinical studies and well-designed randomized-controlled treatment trials present hurdles for researchers and clinicians. MOGAD has transformed from a mysterious entity into a well-defined disorder with targeted diagnostic tools and evolving pathophysiology studies. As we gaze into the future, collaborative research efforts, cutting-edge technology, and a patient-centric approach will likely steer MOGAD toward more accurate and tailored treatments, ultimately improving patient outcomes. The journey to comprehend the complexities of this disorder may be intricate, but the pursuit of unlocking its secrets remains steadfast, driven by the potential to transform lives and redefine our understanding of autoimmune neurology.

## Author contributions

SS: Writing—original draft. LP: Writing—original draft. MM: Writing—original draft.
